# Chip-scale high-performance photonic microwave oscillator

**DOI:** 10.1126/sciadv.ado9570

**Published:** 2024-08-14

**Authors:** Yang He, Long Cheng, Heming Wang, Yu Zhang, Roy Meade, Kerry Vahala, Mian Zhang, Jiang Li

**Affiliations:** ^1^ hQphotonics Inc., 2500 E Colorado Blvd Suite 330, Pasadena CA 91107, USA.; ^2^ HyperLight Corporation, 1 Bow Street, Suite 420, Cambridge, MA 02138, USA.; ^3^ T. J. Watson Laboratory of Applied Physics, California Institute of Technology, Pasadena, CA 91125, USA.

## Abstract

Optical frequency division based on bulk or fiber optics provides unprecedented spectral purity for microwave oscillators. To extend the applications of this approach, the challenges are to develop miniaturized oscillators without trading off phase noise performance. Here, we report a chip-scale high-performance photonic microwave oscillator based on integrated electro-optical frequency division. Dual distributed-feedback lasers are co-self-injection locked to a single silicon nitride spiral resonator to provide a record-high-stability, fully on-chip optical reference. An integrated electro-optical frequency comb based on a thin-film lithium niobate phase modulator chip is leveraged to perform optical-to-microwave frequency division. The resulting integrated photonic microwave oscillator achieves a record-low phase noise for chip-scale oscillators. The results represent a major advance in high-performance, integrated photonic microwave oscillators for applications including signal processing, radar, timing, and coherent communications.

## Teaser

Microwave generation based on integrated photonics provides record-high spectral purity for chip-scale oscillators.

## INTRODUCTION

Low–phase noise microwave oscillators are critically important to a variety of applications including coherent communications, radar systems, high speed digital sampling, test and measurement, and next-generation mobile communications in 5G and 6G. Photonic generation of microwave signals, including optical frequency division (OFD) (*1*), microcombs (*2*), mode-locked laser frequency combs (*3*), optoelectronic oscillators (*4*), and Brillouin oscillators (*5*, *6*), have many attractive properties and advantages over microwave electronics, due to their low phase noise levels, large operating frequency range (up to millimeter wave), and potential for chip-scale packaging. Among the various types of photonic microwave oscillators, OFD has become the preeminent approach for generation of high-performance microwave signals (*7*), offering the lowest phase noise microwave signals at X band (around 12 GHz) (*8*) based on cavity-stabilized lasers and fiber-based frequency combs. A variation of OFD called electro-optical frequency division (eOFD) performs optical-to-microwave frequency division using electro-optical frequency combs (*9*). Compact eOFD oscillators with record-low phase noise at K band (40 GHz) have been demonstrated (*10*), reducing the form factor of high-performance OFD systems to less than 3 liters including all optics and electronics. To date, there have been several demonstrations of OFD and eOFD photonic microwave oscillators (*7*–*18*). To expand the application reach of these signal sources in mobile, airborne, and chip-scale systems, the development of miniaturized chip-scale OFD and eOFD oscillators that reduce size, weight, and power without trading off phase noise performance is of keen interest to the scientific and industrial communities.

Integration of the OFD or eOFD based microwave oscillators requires integration of two key elements: III-V lasers with optical reference cavities, and optical frequency combs. Considering reference cavities first, spiral resonators (*19*–*21*) suppress thermorefractive noise (TRN) through their large mode volumes (*19*). Self-injection locking of a single distributed-feedback (DFB) laser to a 1.4-m-long Si_3_N_4_ spiral reference markedly suppresses TRN noise to a level of −80 dBc/Hz at 10-kHz offset (*20*). Pound-Drever-Hall (PDH) frequency locking of dual external-cavity lasers to on-chip disk microresonators (*9*) and spiral resonators (*17*) has also been shown to provide dual-laser references with additional stability from common-mode rejection, achieving a phase noise level of −80 dBc/Hz at 10-kHz offset based on a 4-m-long Si_3_N_4_-based spiral resonator (*17*). Looking ahead, further improvement of the OFD microwave signal performance and form factor will require copackaged, chip-scale lasers including the III-V gain medium and the reference cavity, with improved spectral purity.

Concerning chip-based frequency combs, soliton microcombs (*2*) generated in a high-*Q* microresonator using the Kerr nonlinearity provide an excellent way to realize on-chip optical-to-microwave frequency division. Recently, soliton microcombs have been used to achieve very low phase noise levels by OFD (*16*, *17*), using the two-point locking approach. In recent years, the thin-film LiNbO_3_ (TFLN) electro-optic combs (EO comb) have also witnessed rapid progresses. Even more generally, TFLN is offering remarkable advances in low-*V*_π_ phase and intensity modulators (*22*–*28*). TFLN EO combs have been generated in ring resonators (*29*, *30*), as well as using nonresonant phase modulators and amplitude modulators (*24*, *31*, *32*). The nonresonant EO comb versions feature tunable repetition rates and rapid tuning speeds, which are desirable in OFD systems for low phase noise generation within a large servo locking bandwidth.

In this work, a chip-scale high-performance photonic microwave oscillator is developed based on a fully on-chip dual-laser reference and an integrated TFLN EO comb. First, the low–phase noise dual-laser reference (including two III-V lasers and on-chip reference cavity) is demonstrated on the basis of co-self-injection locking (cSIL) of two DFB laser chips to a 14-m-long, single Si_3_N_4_ spiral resonator. This dual reference achieves a record-low on-chip optical phase noise (−94 dBc/Hz at 10-kHz offset) and greatly simplifies the OFD system architecture. Specifically, the cSIL reference obviates the need for off-chip components such as optical isolators, external-cavity lasers, and multiple laser frequency locking components, as used in various types of OFD systems. Second, an integrated EO comb is used to perform optical-to-microwave frequency division. The integrated EO comb is generated by pure phase modulation from a thin-film lithium niobate phase modulator. The resulting integrated eOFD oscillator achieves a low phase noise level of −129 dBc/Hz at 10-kHz offset for 37.7-GHz carrier output, which is equivalent to −141 dBc/Hz at 10-kHz offset for a 10-GHz carrier.

## RESULTS

### System design

**Fig. 1.**
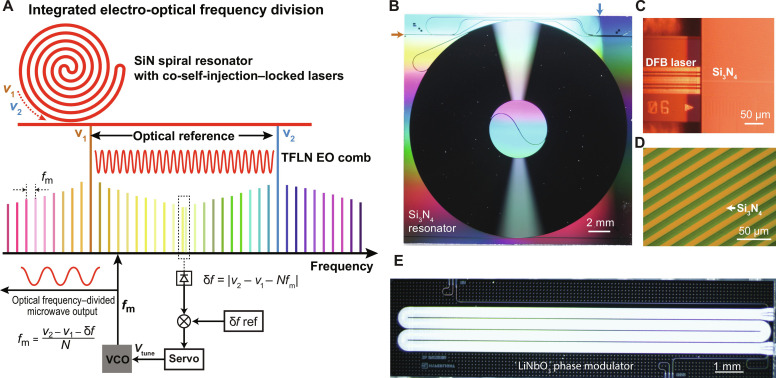
Concept of integrated eOFD. (**A**) Illustration for a photonic microwave oscillator based on integrated eOFD. A fully on-chip dual-laser reference (ν_1_ and ν_2_) is generated by cSIL of dual DFB lasers to an ultrahigh-*Q* Si_3_N_4_ spiral resonator. The dual-laser reference (ν_1_ and ν_2_) is strongly phase modulated using an integrated LiNbO_3_ modulator to generate two electro-optic (EO) combs. At the spectral mid point between ν_1_ and ν_2_, the far upper sideband of ν_1_ nearly overlaps with the far lower sideband of ν_2_. A beat note δ*f* is generated between these two nearly overlapping EO comb lines, which is given by δ*f* = ∣ν_2_ − ν_1_ − *Nf*_m_∣, where *N* is the number of EO comb lines between ν_1_ and ν_2_. This beat note is used for feedback control of the voltage-controlled oscillator (VCO). (**B**) Photograph of the 14-m-long Si_3_N_4_ spiral resonator with footprint 21 by 21 mm is shown. The two input waveguides for butt coupling to two DFB lasers are located on the left edge and the upper edge of the Si_3_N_4_ chip, as indicated by arrows. (**C**) One of the two DFB lasers is shown butt coupled to one input waveguide of the Si_3_N_4_ spiral resonator. The second DFB laser is also butt coupled to the second input waveguide of the Si_3_N_4_ spiral resonator (not shown). (**D**) Microscope image of the Si_3_N_4_ spiral waveguides (yellow color). (**E**) Photograph of a low-*V*_π_ TFLN phase modulator chip with recycled design (double pass) and bent electrodes is shown. Chip footprint is 13.75 by 3.5 mm.

The system concept for the chip-scale photonic microwave oscillator based on integrated eOFD is illustrated in Fig. 1A. A fully chip-scale stable laser reference is generated by cSIL of two DFB lasers to an ultrahigh-*Q* Si_3_N_4_ spiral reference cavity. Besides system simplifications noted above compared with PDH-locked frequency-stabilized lasers, the cSIL approach has the added benefit that relative coherence of the two locked lasers is inherently insensitive to common mode frequency noise of the Si_3_N_4_ spiral cavity. The Si_3_N_4_ spiral resonator (shown in Fig. 1B with a footprint of 21 mm by 21 mm) has a 14-m round-trip length for TRN suppression while achieving a record-high spiral resonator *Q* factor of 332 million. A photograph of one of the DFB lasers butt coupled to one input waveguide of the Si_3_N_4_ spiral resonator is shown in Fig. 1C. Figure 1D shows a microscope image of the Si_3_N_4_ spiral waveguides (yellow color).

The integrated EO comb is generated by phase modulation with a TFLN phase modulator chip, shown in Fig. 1E. The phase modulator incorporates recycled optical waveguides, which double the phase modulator length given a fixed length of coplanar waveguide electrodes. The bent-electrode design increases total electrode length to 50 mm in a small foot print of only 13.75 mm by 3.5 mm. Notably, the recycled optical waveguide design reduces *V*_π_ and drive power for broadband EO comb generation. Compared to microcomb-based approaches, the TFLN EO comb frequency divider does not require a pump laser for microcomb generation, and obviates highly linear ultrafast photodetectors needed in soliton microcomb-based OFD systems, thereby further reducing system complexity (*9*).

### Spiral resonator cSIL operation

**Fig. 2.**
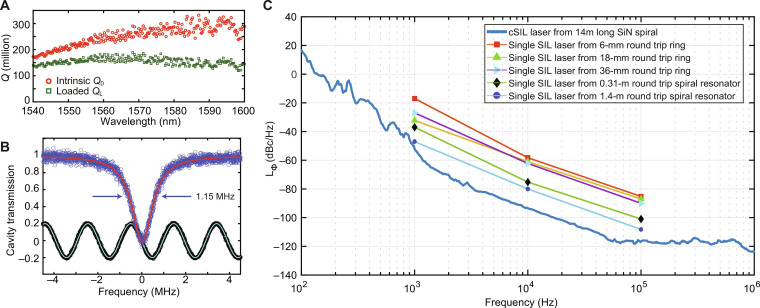
Characterization of the Si_3_N_4_ spiral resonators and the cSIL lasers. (**A**) Measured intrinsic (*Q*_0_, red circle markers) and loaded (*Q*_L_, green square markers) *Q* factors for the 14-m-long Si_3_N_4_ spiral resonator are plotted versus wavelength from 1540 to 1600 nm. (**B**) Cavity transmission of the ultrahigh-*Q* mode at 1587 nm in the 14-m-long Si_3_N_4_ spiral resonator shows a linewidth of 1.15 MHz, loaded *Q*_L_ of 166 million, and intrinsic *Q*_0_ of 332 million. The sine wave is the Mach-Zehder interferometer output for laser frequency calibration. (**C**) Phase noise of dual DFB lasers co-self-injection locked (cSIL) to the ultrahigh-Q, 14-m-long, Si_3_N_4_ spiral resonator (blue curve). The phase noise is independent of the frequency separation of the dual cSIL lasers and is −94 dBc/Hz at 10-kHz offset. For comparison, the phase noise spectra of a single-laser self-injection locked to Si_3_N_4_ ring or spiral resonators with different round trip lengths (6 mm, 18 mm, 36 mm, 0.35 m, and 1.4 m) are shown (*20*, *33*).

The ultrahigh-*Q* Si_3_N_4_ spiral resonators are fabricated at a complementary metal-oxide semiconductor foundry and consist of interleaved (inward and outward) Archimedean spiral waveguides with a pitch of 40 μm. An S-shaped waveguide connects the interleaved spirals with a rotation symmetry of 180°. The ultralow loss Si_3_N_4_ waveguides have a width of 10 μm and a thickness of 100 nm. Limited by the maximum reticle size of 21 mm by 21 mm of the stepper photolithography tool, a maximum round-trip length of 14 m is achieved (see Fig. 1B). The loaded (green markers) and intrinsic *Q* factors (red markers) measured over a wavelength span of 60 nm encompassing C-band are shown in Fig. 2A. Figure 2B shows the Lorentzian fitting for the cavity transmission at 1587 nm, which features a record-high intrinsic *Q* factor of 332 million for spiral resonators. A summary of the measured *Q* factors from other spiral resonators is provided in table S1 of the Supplementary Materials.

To characterize the relative phase noise of DFB lasers under cSIL operation, two DFB lasers with center frequency separation of only 20 GHz were co-self-injection–locked to the spiral resonator. The beat note of the cSIL lasers was then detected on a fast photodetector with bandwidth of 40 GHz, and the phase noise of the 20-GHz beat note was measured (blue curve in Fig. 2D). A relative phase noise of −94 dBc/Hz at 10-kHz offset is measured. For comparison, the absolute phase noise of a single-laser self-injection locked to ultrahigh-*Q* Si_3_N_4_ ring resonators and Si_3_N_4_ spiral resonators having different round-trip lengths is shown in Fig. 2C [data from (*20*, *33*)]. Consistent with the scaling of TRN noise observed in these previous measurements, the cSIL laser phase noise (from 1- to 100-kHz offsets) scales approximately inversely with the cavity volume. Specifically, the 14-m-long Si_3_N_4_ spiral resonator phase noise (−94 dBc/Hz at 10-kHz offset) is suppressed by over 10 dB relative to the phase noise (−80 dBc/Hz at 10-kHz offset) of the 1.4-m-long Si_3_N_4_ spiral resonator in (*20*). Because the cSIL noise results from the beat noise of two lasers, it is expected to be 2× larger than the underlying noise of each laser so that the overall noise is about 17 dB lower in the 14-m cSIL device. As the 14-m spiral resonator results in 10-dB lower TRN noise comparing with the 1.4-m spiral resonator, the cSIL laser noise cancellation locked to a single 14-m spiral resonator is estimated around 7 dB at 10-kHz offset, compared with two lasers injection-locked to two different 14-m spiral resonators. The white–phase noise floor above 60-kHz offset is due to the white thermal noise from the fast photodetector. Comparing the dual-locked laser phase demonstrated here to earlier reports (all at 10-kHz offset), dual on-chip Brillouin laser references have a phase noise of −90 dBc/Hz using a silica disk resonator (*5*) and −84 dBc/Hz using a Si_3_N_4_ ring resonator (*6*) at 10-kHz offset. In addition, dual-laser references by PDH frequency locking of two external-cavity lasers to a Si_3_N_4_ spiral resonator have achieved a phase noise of −80 dBc/Hz (*17*).

### Integrated LiNbO_3_ EO comb generation

A single TFLN phase modulator with low *V*_π_ performance was developed in this work for broadband integrated EO comb generation. For this phase modulator, an electrode length of 50 mm was used, wherein the electrodes bend three times to create four rows of straight electrodes (shown in Fig. 1E). Also, a recycled design for the TFLN optical waveguides was implemented to double the overall electro-optic modulation length to 100 mm. A similar recycled design for the TFLN phase modulator was implemented previously using straight electrodes to double the EO modulation length (*24*). These features reduce *V*_π_ while also maintaining a compact modulator footprint of 13.75 mm by 3.5 mm. Microstructured electrodes were also implemented in this device to improve the modulator frequency response at higher frequencies similar to those demonstrated in (*23*).

**Fig. 3.**
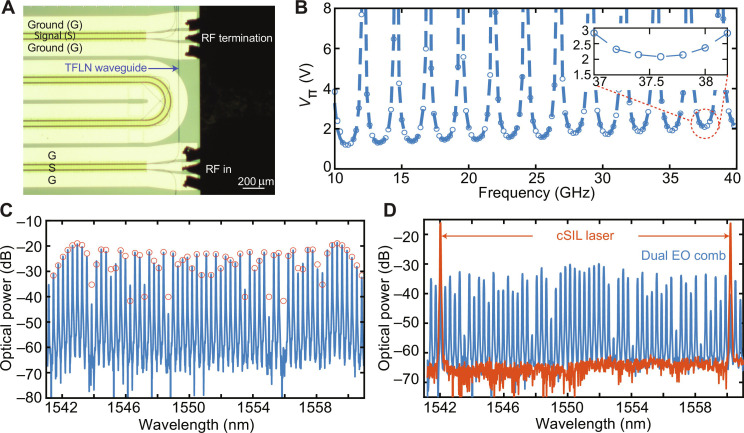
Characterization of the TFLN phase modulator and integrated EO comb generation. (**A**) Microscope image of the TFLN phase modulator shows the RF in and termination using the ground-signal-ground (GSG) microwave probes. The TFLN waveguide is also shown. (**B**) Measured *V*_π_ of the recycled TFLN phase modulator chip with respect to modulation frequency is shown. Inset shows the zoom-in *V*_π_ data around 37.7 GHz. (**C**) Generated broadband EO comb spectrum (37.7-GHz line spacing, 3-dB bandwidth of 2.2 THz) from the TFLN phase modulator chip with a single CW input laser at 1551.1 nm. The circle markers are the theoretical EO comb spectrum for TFLN phase modulator *V*_π_ = 2.1 V and input RF power of 35.9 dBm. (**D**) Optical spectra for the cSIL laser (red curve, 2.26-THz span) and the dual EO comb generated from the TFLN chip (blue curve, 37.7-GHz comb line spacing).

Figure 3A shows the microscope image of the right side of the TFLN chip. A dual radio frequency (RF) probe with ground-signal-ground configuration was used to launch microwave signals into the input electrode and terminate the RF electrode end at the output electrode. The measured *V*_π_ of the modulator plotted versus modulation frequency is shown in Fig. 3B. Because of the recycled design, the in-phase superposition of the phase modulation between the first pass and the second pass causes a 2× reduction of the *V*_π_ at frequencies (*N* + 1/2)FSR, where FSR is the free spectral range, and *N* is an integer. The measured *V*_π_ is 1.5 V at 18 GHz and 2.1 V at 40 GHz. To the best of our knowledge, these are record-low *V*_π_ values for a LiNbO_3_ phase modulator at these frequencies for telecomm C-band wavelengths. For comparison, the two-pass, recycled TFLN phase modulator in (*24*) has a *V*_π_ of 2.6 V at 18.5 GHz. Also, a quad-pass recycled TFLN phase modulator has a *V*_π_ of 2.5 V at 20 GHz (*32*). Table S2 in the Supplementary Materials summarizes *V*_π_ measurements from various LiNbO_3_ phase modulators at C-band wavelengths. The resulting EO comb with 37.7-GHz line spacing and 2.2-THz bandwidth (3 dB) (launched RF power of 36 dBm) is shown in Fig. 3C, with a single continuous wave laser input at 1551 nm. The circle markers in Fig. 3C are the theoretical EO comb spectrum with a fitted phase modulation depth δϕ = π*V*/*V*_π_ = 29.4, which corresponds to an input RF power of 35.9 dBm and *V*_π_ = 2.1 V at 37.7 GHz. The measured EO comb spectrum is in excellent agreement with the theoretical spectrum. Figure 3D shows the optical spectra for the cSIL laser with 2.26-THz frequency separation (red curve), as well as the dual EO comb (blue curve) generated from the TFLN phase modulator chip.

### Oscillator noise performance

**Fig. 4.**
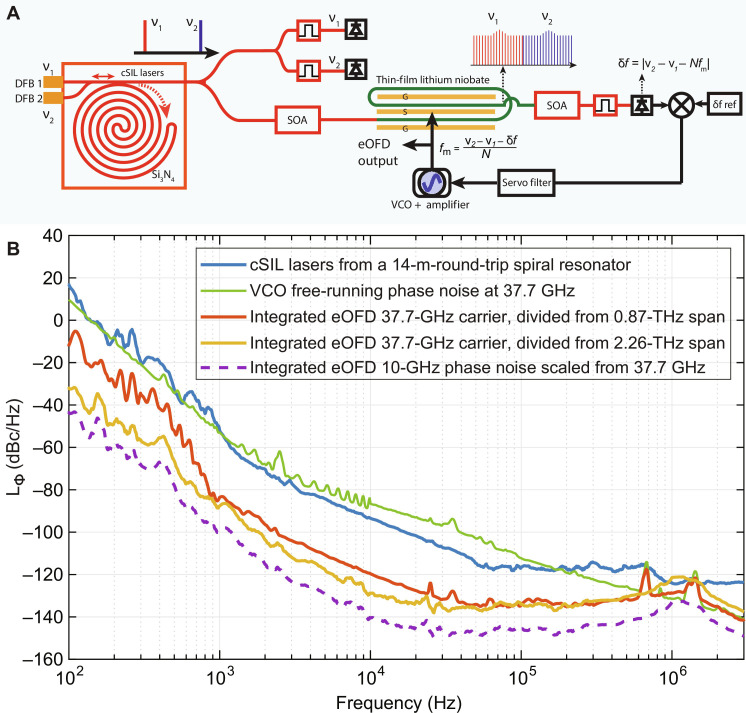
Experimental setup and phase noise measurement of the chip-scale eOFD oscillator. (**A**) Two DFB lasers are co-self-injection locked (cSIL) to an ultrahigh-*Q* Si_3_N_4_ spiral resonator. A 50/50 Si_3_N_4_ waveguide directional coupler combines the two laser inputs before coupling to the spiral resonator. The cSIL laser references are then coupled out at the Si_3_N_4_ through port and amplified by a semiconductor optical amplifier (SOA). After the SOA, the cSIL lasers are coupled to the TFLN low-*V*_π_ phase modulator chip using a lensed fiber to generate on-chip EO combs with line spacing of 37.7 GHz. The spectral middle point lines between dual reference lasers are optically band-pass filtered and detected for amplified phase error detection of the EO comb line spacing phase noise. The eOFD phase error is then used for feedback control of the VCO via a fast servo filter. (**B**) Phase noise of the chip-scale eOFD oscillator is shown. The blue curve is the reference phase noise of the cSIL dual-laser reference from the 14-m-long Si_3_N_4_ spiral resonator. The green curve is the free-running phase noise of the VCO at 37.7 GHz. The red (yellow) curve is the phase noise of the chip-scale eOFD oscillator at 37.7-GHz carrier, divided from 0.87 (2.26) THz cSIL dual-laser span. A phase noise level of −129 dBc/Hz at 10-kHz offset was obtained for the 37.7-GHz carrier (yellow curve). The dashed purple curve is the phase noise scaled to a 10-GHz carrier frequency from the 37.7-GHz output (vertical scale offset by 20log_10_[37.7 GHz/10 GHz]), showing an equivalent phase noise of −141 dBc/Hz at 10-kHz offset.

Figure 4A shows the experimental setup for the chip-scale eOFD oscillator. Two cSIL DFB lasers at wavelengths 1542 and 1560 nm are used to create a 2.26-THz frequency span optical reference. A 50/50 Si_3_N_4_ waveguide directional coupler combines the two input ports before coupling to the spiral resonator using a pulley coupler design. The cSIL laser signals are then coupled out at the Si_3_N_4_ through port (∼2 mW) and amplified by a semiconductor optical amplifier (SOA) to 25 mW. After the SOA, the cSIL signals are coupled to the TFLN low-*V*_π_ phase modulator chip using a lensed fiber. The resulting EO combs (with line spacing 37.7 GHz, shown in Fig. 3D) overlap at the spectral middle point between the cSIL laser lines. The EO combs are postamplified using a second SOA to 5 mW. The spectral middle point lines between dual reference lasers are optically band-pass filtered and detected. The eOFD phase error is then generated and used for feedback control of the voltage-controlled oscillator (VCO) via a fast servo filter. The optical-to-microwave frequency division factor of *N* = 60 results in a phase noise reduction of 35 dB (= 20log_10_N) from the optical reference to the microwave carrier at 37.7 GHz. The measured phase noise spectrum of the 37.7-GHz carrier is presented in Fig. 4B (yellow curve). A phase noise level of −129 dBc/Hz at 10-kHz offset, and −136 dBc/Hz at 50-kHz offset is obtained for the 37.7-GHz carrier.

For comparison, the phase noise of the cSIL optical reference signal is also shown in the figure (blue curve) as well as the free-running phase noise of the VCO at 37.7 GHz (green curve). Here, we used a tunable yttrium-iron-garnet VCO to tune to the minimum *V*π frequency of the TFLN modulator and for the eOFD operation. Also, the higher operation frequency of 37.7 GHz was used in the experiment to achieve a broader EO comb bandwidth compared with operating at lower modulation frequencies (i.e., narrower EO comb line spacing). A second eOFD measurement using cSIL lasers with a reduced frequency span of 0.87 THz (but same comb line spacing of 37.7 GHz) is also shown (red curve, division factor *N* = 23). For offset frequencies less than ∼30 kHz, the phase noise of the eOFD microwave signal phase noise is determined by the divided phase noise (by 20log_10_N dB) from the optical reference phase noise. For offsets above 40 kHz, the phase noise of the eOFD microwave signal phase noise is determined by the suppression of the free-running VCO phase noise. See Materials and Methods for detailed discussions. The dashed purple curve in Fig. 4B is the phase noise scaled to a 10-GHz carrier frequency (vertical scale offset by 20log_10_ [37.7 GHz/10 GHz]), showing an equivalent phase noise of −141 dBc/Hz at 10-kHz offset and −148 dBc/Hz at 50-kHz offset. The 10-kHz offset phase noise level of −141 dBc/Hz for 10-GHz carrier is a record-low phase noise for chip-scale photonic microwave oscillators and on par with the recent demonstration using a photonic-chip-based, soliton microcomb OFD system (*16*).

## DISCUSSION

**Fig. 5.**
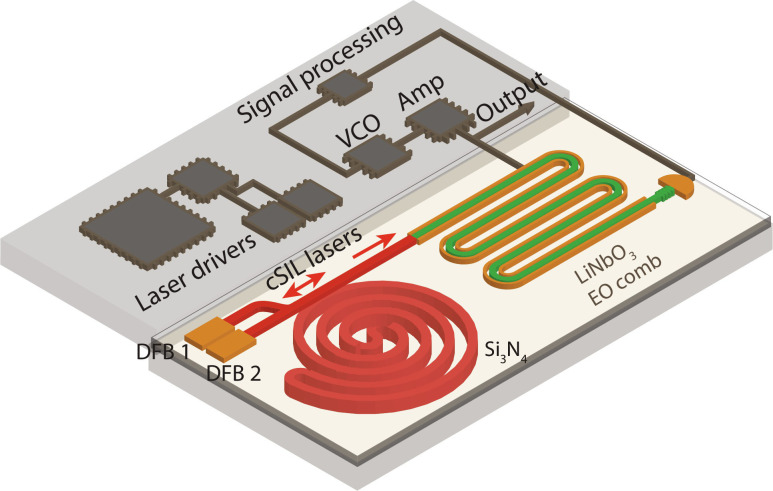
Design for an integrated multichip-module photonic microwave oscillator. The integrated system incorporates the same chip-scale photonic devices in this work. A fully on-chip dual-laser reference is realized by cSIL of dual DFB lasers to an ultrahigh-*Q* spiral resonator. A TFLN EO comb is generated for optical-to-microwave frequency division.

Figure 5 shows the design for a multichip-module photonic microwave oscillator. The fully integrated oscillator system incorporates the two DFB lasers, Si_3_N_4_ spiral resonator, TFLN modulator, and InGaAs photodetector. Recent advances based on hybrid integration and heterogeneous integration of multiphotonic chips can be leveraged for scalable production of the oscillator. For example, hybrid integration based on efficient butt coupling between the DFB pump laser and a high-*Q* Si_3_N_4_ ring resonator has been demonstrated for compact turn-key soliton generation (*34*). Heterogeneous integration of III-V with Si_3_N_4_ has also shown with low mode-transition losses (<1 dB) (*35*, *36*). Heterogeneous integration between TFLN and Si_3_N_4_ waveguides has been demonstrated with <1 dB mode transition loss (*37*, *38*). Also, a bilayer inverse taper design has been used to convert the strongly confined TFLN ridge waveguide mode to a weakly confined TFLN slab mode, which provides low interface loss (<1.7 dB) for butt coupling between the TFLN facet and a lensed fiber (*39*). The similar inverse taper design of the TFLN mode converter is expected to provide efficient power coupling to the slab mode of the SiN waveguide. The two SOAs used in the current demonstration can be omitted in future designs by increasing cSIL laser power and by reducing the Si_3_N_4_ to TFLN coupling loss and the TFLN on-chip propagation loss. Additional components in the multichip module shown in Fig. 5 include integrated laser driver circuits, an integrated VCO and a power amplifier for EO comb generation and eOFD.

Moreover, the current integrated eOFD demonstration used a 2.26-THz frequency span reference, limited by the nonresonant TFLN EO comb optical bandwidth. Recent EO combs based on TFLN ring resonators produce much broader EO comb bandwidths (*29*, *30*). Integrated eOFD with wider resonant EO combs would lead to additional >10-dB phase noise reductions by further increasing the optical-to-microwave division ratio. Also, the current work uses a TFLN phase modulator chip with a double-pass design to reduce the *V*_π_ of the modulator, resulting in periodic changes in the *V*_π_ with respect to the modulation frequency. This can be mitigated by incorporating two or more phase modulators (each with a single-pass design) in series. The frequency response of the single-pass phase modulator is monotonic with modulation frequency, which is desirable for increasing the frequency tunability of the chip-scale eOFD microwave signal source.

In conclusion, a chip-scale photonic microwave oscillator with record-low phase noise has been reported. The oscillator implements integrated eOFD with two important chip-scale device innovations. First, a fully on-chip, dual optical reference is demonstrated on the basis of a monolithic ultrahigh-*Q* silicon nitride spiral resonator operated in co-self-injection–locking mode with two DFB lasers. This cSIL demonstration markedly simplifies the frequency reference by eliminating various off-chip components such as optical isolators, external-cavity lasers, and laser frequency locking components required in other OFD systems. Moreover, it provides record-low phase noise for on-chip lasers through the record-high-*Q* and large-mode-volume spiral design. Second, an integrated low-*V*_π_ phase modulator based on a recycled, dual-pass geometry design enables wide EO comb bandwidth at reduced drive power. The simplified architecture eliminates several subsystems and components that are typically required in OFD systems, thereby reducing complexity and ultimate system size. Also, the mass-producible, solid-state optical reference demonstrated here improves robustness and manufacturing economy of scale. The high-performance, chip-scale photonic microwave oscillator demonstrated here represents a major advance in integrated photonic microwave oscillators and is expected to have notable performance impacts on many applications including precision timing, signal processing, radar, and coherent communications.

## MATERIALS AND METHODS

The Si_3_N_4_ spiral resonator is based on the low-confinement, high–aspect ratio (10-μm-wide, 100-nm-thick Si_3_N_4_ core) Si_3_N_4_ waveguides, fabricated in a complementary metal-oxide semiconductor (COMS)-ready foundry (*20*, *33*). The detailed fabrication processes are described in (*33*). The Si_3_N_4_ core is cladded by thermal SiO_2_ (lower cladding) and LPCVD SiO_2_ (upper cladding). An on-chip Si_3_N_4_ directional coupler with 50/50 coupling ratio is placed on the input bus waveguide, to combine the two DFB laser inputs before the Si_3_N_4_ spiral resonator.

For Si_3_N_4_ spiral resonator *Q* measurement, a continuously-tunable, external-cavity diode laser (Toptica CTL 1550) was used to scan across the resonator resonance frequencies. The instantaneous, short-term linewidth of the scanning laser is <10 kHz, much smaller than the resonator linewidth (∼1 MHz) in our measurements. The frequency scan range of the laser was calibrated and measured at the same time using a separate Mach-Zehnder interferometer while the laser was scanning across the resonator resonances.

For cSIL, the first DFB laser was butt coupled to one input Si_3_N_4_ waveguide on the left side of the Si_3_N_4_ chip, while the second DFB laser was butt coupled to the second input waveguide on the top edge of the Si_3_N_4_ chip (shown in Fig. 1, B and C). A 50/50 directional coupler was placed on the same Si_3_N_4_ chip to combine the two input waveguides before coupling to the spiral resonator. The resonant back scattering power from the Si_3_N_4_ spiral resonator back to the laser chip was measured ∼1%, relative to the input power of each DFB laser. Self-injection locking of each DFB laser was achieved by adjusting the butt-coupling gap between the DFB laser and the SiN chip using a precision translation stage and monitoring the resonator through port power on a photodetector. The DFB lasers are commercial laser chips from PhotonX. In addition, the DFB laser operation points used in the experiments are as follows: laser temperature 21.8° to 30.5°C, current 170 mA, and output power 55 mW.

For TFLN device fabrication, a commercial x-cut LN-on-insulator wafer (NANOLN) was used. On the wafer, a TFLN layer was on top of a SiO_2_/Si stack substrate. Deep UV (DUV) and Ar+ based reactive ion etching were used to define the optical waveguides in the TFLN. The TFLN waveguide top ridge width was 2 μm. The entire device was cladded with silicon dioxide via plasma-enhanced chemical vapor deposition. Then, metal electrodes were patterned using a self-aligning lift-off process. The ground-signal gap was ∼5 μm (inner edge to inner edge). In the end, the chip edges were diced for fiber to chip coupling. The optical group index for the TE_0_ mode was 2.19 (simulated by Lumerical), and the measured RF effective index for the electrodes was 2.16, therefore achieving good velocity matching between the optical and RF fields.

For *V*_π_ measurement of the TFLN phase modulator chip, a high-resolution optical spectrum analyzer was used to measure the first-order EO sideband power relative to zeroth order laser power under EO phase modulation, from which the phase modulation depth (δϕ = π*V*/*V*_π_) and the modulator *V*_π_ were calculated at each microwave modulation frequency (*f*_M_) according to the Jacobi-Anger expansion of phase modulated electrical fields

$$E_{0}e^{i\omega_{0}t}e^{i\delta \phi \text{cos}\left(2\pi f_{M}t\right)}=E_{0}e^{i\omega_{0}t}\sum_{n=-\infty }^{+\infty }‍i^{n}J_{n}\left(\delta \phi \right)e^{\text{in}2\pi f_{M}t}$$ (1)

where ω_0_ is the laser carrier angular frequency and *J_n_*(δϕ) is the *n*th Bessel function of the first kind.

A lensed fiber was used to couple light out from the Si_3_N_4_ spiral resonator chip with an edge coupling loss of 2 dB. Two lensed fibers were used to couple light into and out from the TFLN chip with a total fiber-chip-fiber insertion loss of 13 dB. The lensed fiber to TFLN edge coupling loss is measured at 4 dB per facet. Therefore, the on-chip propagation loss for the TFLN phase modulator is 5 dB.

For the phase noise measurement, an ultralow phase noise 40-GHz eOFD oscillator with a compact modular form factor described in (*10*) was used to down-convert the 37.7-GHz chip-eOFD oscillator signal to 2.3 GHz. The phase noise of the 2.3-GHz signal was then measured by a commercial phase noise analyzer (Rohde Schwarz FSUP26). A standard smoothing option was used by the phase noise analyzer with cross correlations number 100 and measurement time of 14.7 s. Also, spurs were not plotted in the phase noise spectrum. The phase noise of the reference 40-GHz eOFD oscillator (−153 dBc/Hz at 10-kHz offset for 40-GHz carrier) is much lower than the phase noise of the chip-scale eOFD oscillator.

The phase noise of the eOFD oscillator is given by (*9*)

$$S_{\phi }^{\text{eOFD}}\left(f\right)=\frac{{|G_{\text{op}}\left(f\right)|}^{2}}{{|1+NG_{\text{op}}\left(f\right)|}^{2}}S_{\phi }^{\text{ref}}\left(f\right)+\frac{1}{{|1+NG_{\text{op}}\left(f\right)|}^{2}}S_{\phi }^{\text{VCOfr}}\left(f\right)$$ (2)

where *G*_op_(*f*) is the open loop transfer function for the eOFD servo loop (*9*), *N* is the optical-to-microwave frequency division ratio, $S_{\phi }^{\text{ref}}\left(f\right)$ is the phase noise spectrum of the dual optical frequency reference, and $S_{\phi }^{\text{VCOfr}}\left(f\right)$ is the phase noise of the free-running VCO and amplifier. For offset frequency much lower than the eOFD locking bandwidth (∼1 MHz), *NG*_op_ ≫ 1, and for offset frequency much greater than the eOFD locking bandwidth (∼1 MHz), *NG*_op_ ≪ 1. The first term in Eq. 2 denotes phase noise divided by *N*^2^ from the optical reference due to optical-to-microwave frequency division, while the second term acts as a high-pass filter to suppress the free-running phase noise of the VCO within the locking bandwidth.

## Supplementary Materials

### This PDF file includes:

Fig. S1 Tables S1 and S2 References
