# Supplementary material for: Chip-scale high-performance photonic microwave oscillator
Source: Sci Adv. 2024 Aug 14;10(33):eado9570. doi: 10.1126/sciadv.ado9570 (PMC11323879; doi:10.1126/sciadv.ado9570)
Supplement: Supplementary file 1 — Fig. S1 Tables S1 and S2 References [file sciadv.ado9570_sm.pdf]

Science Advances
AAAS

Supplementary Materials for

Chip-scale high-performance photonic microwave oscillator

Yang He *et al.*

Corresponding author: Jiang Li, jiang.li@hqphotonics.net

Sci. Adv. **10**, eado9570 (2024)
DOI: 10.1126/sciadv.ado9570

This PDF file includes:

Fig. S1
Tables S1 and S2
References

Single pass versus double pass (recyled) TFLN phase modulator design

The schematics for single-pass and double-pass (recycled) thin film lithium niobate (TFLN) phase modulators are shown in Figure S1. For the single-pass design, the optical waveguide (green line) only passes through the gap between the signal and ground electrodes of the coplanar-waveguide (CPW) once. For the double-pass (recycled) design, after the first pass through the first ground-signal gap of the CPW, the waveguide is looped back to pass through the second ground-signal gap of the CPW. Therefore, the EO modulation depth is doubled for the double-pass (recyled) design, and the V_π is reduced by a factor of two, for microwave modulation frequencies at (N+1/2)FSR, where N is an integer and FSR is the free-spectral-range of

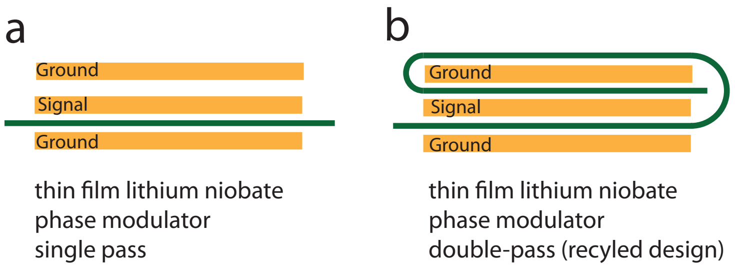

Figure S1: **Schematics for single pass and double pass TFLN phase modulators.** (a) Schematic for a single pass thin film lithium niobate phase modulator. The TFLN waveguide (green line) only passes through the gap between the signal and ground electrodes (yellow) of the coplanar-waveguide (CPW) once. (b) Schematic for a double-pass (recycled design) thin film lithium niobate phase modulator. The TFLN waveguide (green line) passes through the two gaps of the CPW twice.

the recycled phase modulator. The 1/2 term is due to the different electrical field directions for the two CPW gaps.

Summary of various ultra-high-Q spiral resonators

The summary of various ultra-high-Q (UHQ) spiral resonators (*19–21*) is given in Table S1, along with various UHQ Si_3N_4 ring resonators (*33, 40, 41*). The Si_3N_4 spiral resonator in this work achieved an intrinsic Q factor of 332 million, which is a record high Q factor for on-chip spiral resonators. For comparison, the TE mode Si_3N_4 spiral resonator in (*20*) has an intrinsic Q factor of 164 million, and the TM mode Si_3N_4 spiral resonator in (*21*) has an intrinsic Q factor of 80 million. The silica wedge spiral resonator has an an intrinsic Q factor of 140 million (*19*). Note that the high-confinement Si_3N_4 ring resonators (with thicker Si_3N_4 core), with intrinsic Q factors of 30 million (*42*), 67 million (*43*), are not included in the table.

Resonator type	Intrinsic Q Factor Q_0	Round trip length	Mode
SiN spiral resonator [This work]	332 M at 1587 nm	14 m	TE
SiN spiral resonator (*20*)	164 M at 1550 nm	1.4 m	TE
SiN spiral resonator (*21*)	80 M at 1550 nm	4 m	TM
SiO_2 spiral resonator (*19*)	140 M at 1550 nm	1.2 m	TE
SiN ring resonator (*33*)	260 M at 1600 nm	6 mm	TE
SiN ring resonator (*40*)	720 M at 1615 nm	74 mm	TM
SiN ring resonator (*41*)	422 M at 1570 nm	74 mm	TE

Table S1: **Summary of various ultra-high-Q (UHQ) spiral resonators (*19–21*) and Si_3N_4 ring resonators (*33, 40, 41*).**

Phase Modulator Type	V_π	Architecture
TFLN phase modulator [This work]	1.5 V at 18 GHz<br>1.6 V at 25.5 GHz	Recycled TFLN<br>dual pass
TFLN phase modulator (*32*)	2.5 V at 20 GHz<br>2.0 V at 25 GHz	Recycled TFLN<br>quad pass
TFLN phase modulator (*24*)	2.6 V at 18.5 GHz<br>2.3 V at 21.5 GHz	Recycled TFLN<br>dual pass
TFLN phase modulator (*31*)	4.1 V at 20 GHz	Single pass TFLN
Commercial phase modulator (*44*)	3.8 V at 18 GHz	Single pass bulk LN
Commercial phase modulator (*45*)	4.0 V at 18 GHz	Single pass bulk LN

Table S2: **Summary of the V_π and architecture of various $LiNbO_3$ (LN) phase modulators.**

Summary of various low V_π phase modulators

Table S2 shows the summary of V_π and architecture of various thin film $LiNbO_3$ phase modulators (*24, 31, 32*), and commerical bulk $LiNbO_3$ phase modulators (*44, 45*). The TFLN phase modulator chip developed in this work features a measured V_π of 1.5V at 18 GHz, and V_π of 1.6V at 25.5 GHz, which is the record-low V_π for $LiNbO_3$ phase modulators at telecomm C-band wavelength. Note that various low V_π TFLN intensity modulators are not included in Table S2, as intensity modulators are not used for broadband EO comb generation in this work.

REFERENCES AND NOTES

1. S. A. Diddams, K. Vahala, T. Udem, Optical frequency combs: Coherently uniting the electromagnetic spectrum. *Science* **369**, eaay3676 (2020).

2. T. J. Kippenberg, A. L. Gaeta, M. Lipson, M. L. Gorodetsky, Dissipative Kerr solitons in optical microresonators. *Science* **361**, doi: 10.1126/science.aan8083 (2018).

3. M. Kalubovilage, M. Endo, T. R. Schibli, X-band photonic microwaves with phase noise below- 180 dbc/hz using a free-running monolithic comb. *Opt. Express* **30**, 11266–11274 (2022).

4. L. Maleki, The optoelectronic oscillator. *Nat. Photonics* **5**, 728–730 (2011).

5. J. Li, H. Lee, K. J. Vahala, Microwave synthesizer using an on-chip brillouin oscillator. *Nat. Commun.* **4**, 2097 (2013).

6. S. Gundavarapu, G. M. Brodnik, M. Puckett, T. Huffman, D. Bose, R. Behunin, J. Wu, T. Qiu, C. Pinho, N. Chauhan, J. Nohava, P. T. Rakich, K. D. Nelson, M. Salit, D. J. Blumenthal, Sub-hertz fundamental linewidth photonic integrated Brillouin laser. *Nat. Photonics* **13**, 60–67 (2019).

7. T. M. Fortier, M. S. Kirchner, F. Quinlan, J. Taylor, J. Bergquist, T. Rosenband, N. Lemke, A. Ludlow, Y. Jiang, C. Oates, S. A. Diddams, Generation of ultrastable microwaves via optical frequency division. *Nat. Photonics* **5**, 425–429 (2011).

8. X. Xie, R. Bouchand, D. Nicolodi, M. Giunta, W. Hänsel, M. Lezius, A. Joshi, S. Datta, C. Alexandre, M. Lours, P.-A. Tremblin, G. Santarelli, R. Holzwarth, Y. L. Coq, Photonic microwave signals with zeptosecond-level absolute timing noise. *Nat. Photonics.* **11**, 44–47 (2017).

9. J. Li, X. Yi, H. Lee, S. A. Diddams, K. J. Vahala, Electro-optical frequency division and stable microwave synthesis. *Science* **345**, 309–313 (2014).

10. J. Li, K. Vahala, Small-sized, ultra-low phase noise photonic microwave oscillators at x-ka bands. *Optica* **10**, 33–34 (2023).

11. W. C. Swann, E. Baumann, F. R. Giorgetta, N. R. Newbury, Microwave generation with low residual phase noise from a femtosecond fiber laser with an intracavity electro-optic modulator. *Opt. Express* **19**, 24387–24395 (2011).

12. A. Ishizawa, T. Nishikawa, T. Goto, K. Hitachi, T. Sogawa, H. Gotoh, Ultralow-phase-noise millimetre-wave signal generator assisted with an electro-optics-modulator-based optical frequency comb. *Sci. Rep.* **6**, 24621 (2016).

13. J. Li, K. Vahala, Optical frequency divider based on an electro-optical-modulator frequency comb. U.S. Patent 9,905,999 (2018).

14. I. S. Kudelin, W. Groman, M. Kelleher, D. Lee, A. Lind, C. Mclemore, F. Quinlan, S. Diddams, *Terahertz, RF, Millimeter, and Submillimeter-Wave Technology and Applications XVI* (SPIE, 2023), p. PC124200M.

15. Y. Zhao, J. K. Jang, G. J. Beals, K. J. McNulty, X. Ji, Y. Okawachi, M. Lipson, A. L. Gaeta, All-optical frequency division on-chip using a single laser. *Nature* **627**, 546–552 (2024).

16. I. Kudelin, W. Groman, Q.-X. Ji, J. Guo, M. L. Kelleher, D. Lee, T. Nakamura, C. A. McLemore, P. Shirmohammadi, S. Hanifi, H. Cheng, N. Jin, L. Wu, S. Halladay, Y. Luo, Z. Dai, W. Jin, J. Bai, Y. Liu, W. Zhang, C. Xiang, L. Chang, V. Iltchenko, O. Miller, A. Matsko, S. M. Bowers, P. T. Rakich, J. C. Campbell, J. E. Bowers, K. J. Vahala, F. Quinlan, S. A. Diddams, Photonic chip-based low-noise microwave oscillator. *Nature* **627**, 534–539 (2024).

17. S. Sun, B. Wang, K. Liu, M. W. Harrington, F. Tabatabaei, R. Liu, J. Wang, S. Hanifi, J. S. Morgan, M. Jahanbozorgi, Z. Yang, S. M. Bowers, P. A. Morton, K. D. Nelson, A. Beling, D. J. Blumenthal, X. Yi, Integrated optical frequency division for microwave and mmwave generation. *Nature* **627**, 540–545 (2024).

18. Y. Liu, D. Lee, T. Nakamura, N. Jin, H. Cheng, M. L. Kelleher, C. A. McLemore, I. Kudelin, W. Groman, S. A. Diddams, P. T. Rakich, F. Quinlan, Low-noise microwave generation with an air-gap optical reference cavity. *APL Photonics* **9**, 010806 (2024).

19. H. Lee, M.-G. Suh, T. Chen, J. Li, S. A. Diddams, K. J. Vahala, Spiral resonators for on-chip laser frequency stabilization. *Nat. Commun.* **4**, 2468 (2013).

20. B. Li, W. Jin, L. Wu, L. Chang, H. Wang, B. Shen, Z. Yuan, A. Feshali, M. Paniccia, K. J. Vahala, J. E. Bowers, Reaching fiber-laser coherence in integrated photonics. *Opt. Lett.* **46**, 5201–5204 (2021).

21. K. Liu, N. Chauhan, J. Wang, A. Isichenko, G. M. Brodnik, P. A. Morton, R. O. Behunin, S. B. Papp, D. J. Blumenthal, 36 Hz integral linewidth laser based on a photonic integrated 4.0 m coil resonator. *Optica* **9**, 770–775 (2022).

22. C. Wang, M. Zhang, X. Chen, M. Bertrand, A. Shams-Ansari, S. Chandrasekhar, P. Winzer, M. Lončar, Integrated lithium niobate electro-optic modulators operating at cmos-compatible voltages. *Nature* **562**, 101–104 (2018).

23. P. Kharel, C. Reimer, K. Luke, L. He, M. Zhang, Breaking voltage–bandwidth limits in integrated lithium niobate modulators using micro-structured electrodes. *Optica* **8**, 357–363 (2021).

24. M. Yu, D. Barton III, R. Cheng, C. Reimer, P. Kharel, L. He, L. Shao, D. Zhu, Y. Hu, H. R. Grant, L. Johansson, Y. Okawachi, A. L. Gaeta, M. Zhang, M. Lončar, Integrated femtosecond pulse generator on thin-film lithium niobate. *Nature* **612**, 252–258 (2022).

25. M. Xu, Y. Zhu, F. Pittalà, J. Tang, M. He, W. C. Ng, J. Wang, Z. Ruan, X. Tang, M. Kuschnerov, L. Liu, S. Yu, B. Zheng, X. Cai, Dual-polarization thin-film lithium niobate in-phase quadrature modulators for terabit-per-second transmission. *Optica* **9**, 61–62 (2022).

26. A. N. R. Ahmed, S. Shi, A. Mercante, S. Nelan, P. Yao, D. W. Prather, High-efficiency lithium niobate modulator for k band operation. *Apl Photonics* **5**, 091302 (2020).

27. S. Xue, Z. Shi, J. Ling, Z. Gao, Q. Hu, K. Zhang, G. Valentine, X. Wu, J. Staffa, U. A. Javid, Q. Lin, Full-spectrum visible electro-optic modulator. *Optica* **10**, 125–126 (2023).

28. F. Valdez, V. Mere, S. Mookherjea, 100 GHz bandwidth, 1 volt integrated electro-optic Mach–Zehnder modulator at near-IR wavelengths. *Optica* **10**, 578–584 (2023).

29. M. Zhang, B. Buscaino, C. Wang, A. Shams-Ansari, C. Reimer, R. Zhu, J. M. Kahn, M. Lončar, Broadband electro-optic frequency comb generation in a lithium niobate microring resonator. *Nature* **568**, 373–377 (2019).

30. Y. Hu, M. Yu, B. Buscaino, N. Sinclair, D. Zhu, R. Cheng, A. Shams-Ansari, L. Shao, M. Zhang, J. M. Kahn, M. Lončar, High-efficiency and broadband on-chip electro-optic frequency comb generators. *Nat. Photonics* **16**, 679–685 (2022).

31. T. Ren, M. Zhang, C. Wang, L. Shao, C. Reimer, Y. Zhang, O. King, R. Esman, T. Cullen, M. Lončar, An integrated low-voltage broadband lithium niobate phase modulator. *IEEE Photon. Technol. Lett.* **31**, 889–892 (2019).

32. K. Zhang, W. Sun, Y. Chen, H. Feng, Y. Zhang, Z. Chen, C. Wang, A power-efficient integrated lithium niobate electro-optic comb generator. *Commun. Phys.* **6**, 17 (2023).

33. W. Jin, Q.-F. Yang, L. Chang, B. Shen, H. Wang, M. A. Leal, L. Wu, M. Gao, A. Feshali, M. Paniccia, K. J. Vahala, J. E. Bowers, Hertz-linewidth semiconductor lasers using cmos-ready ultra-high-*Q* microresonators. *Nat. Photonics* **15**, 346–353 (2021).

34. B. Shen, L. Chang, J. Liu, H. Wang, Q.-F. Yang, C. Xiang, R. N. Wang, J. He, T. Liu, W. Xie, J. Guo, D. Kinghorn, L. Wu, Q.-X. Ji, T. J. Kippenberg, K. Vahala, J. E. Bowers, Integrated turnkey soliton microcombs. *Nature* **582**, 365–369 (2020).

35. C. Xiang, J. Guo, W. Jin, L. Wu, J. Peters, W. Xie, L. Chang, B. Shen, H. Wang, Q.-F. Yang, D. Kinghorn, M. Paniccia, K. J. Vahala, P. A. Morton, J. E. Bowers, High-performance lasers for fully integrated silicon nitride photonics. *Nat. Commun.* **12**, 6650 (2021).

36. C. Xiang, W. Jin, O. Terra, B. Dong, H. Wang, L. Wu, J. Guo, T. J. Morin, E. Hughes, J. Peters, Q.-X. Ji, A. Feshali, M. Paniccia, K. J. Vahala, J. E. Bowers, 3d integration enables ultralow-noise isolator-free lasers in silicon photonics. *Nature* **620**, 78–85 (2023).

37. L. Chang, M. H. Pfeiffer, N. Volet, M. Zervas, J. D. Peters, C. L. Manganelli, E. J. Stanton, Y. Li, T. J. Kippenberg, J. E. Bowers, Heterogeneous integration of lithium niobate and silicon nitride waveguides for wafer-scale photonic integrated circuits on silicon. *Opt. Lett.* **42**, 803–806 (2017).

38. M. Churaev, R. N. Wang, A. Riedhauser, V. Snigirev, T. Blésin, C. Möhl, M. H. Anderson, A. Siddharth, Y. Popoff, U. Drechsler, D. Caimi, S. Hönl, J. Riemensberger, J. Liu, P. Seidler, T. J. Kippenberg, A heterogeneously integrated lithium niobate-on-silicon nitride photonic platform. *Nat. Commun.* **14**, 3499 (2023).

39. L. He, M. Zhang, A. Shams-Ansari, R. Zhu, C. Wang, L. Marko, Low-loss fiber-to-chip interface for lithium niobate photonic integrated circuits. *Opt. Lett.* **44**, 2314–2317 (2019).

40. K. Liu, N. Jin, H. Cheng, N. Chauhan, M. W. Puckett, K. D. Nelson, R. O. Behunin, P. T. Rakich, D. J. Blumenthal, Ultralow 0.034 dB/m loss wafer-scale integrated photonics realizing 720 million Q and 380 μw threshold brillouin lasing. *Opt. Lett.* **47**, 1855–1858 (2022).

41. M. W. Puckett, K. Liu, N. Chauhan, Q. Zhao, N. Jin, H. Cheng, J. Wu, R. O. Behunin, P. T. Rakich, K. D. Nelson, D. J. Blumenthal, 422 Million intrinsic quality factor planar integrated all-waveguide resonator with sub-MHz linewidth. *Nat. Commun.* **12**, 934 (2021).

42. J. Liu, G. Huang, R. N. Wang, J. He, A. S. Raja, T. Liu, N. J. Engelsen, T. J. Kippenberg, High-yield, wafer-scale fabrication of ultralow-loss, dispersion-engineered silicon nitride photonic circuits. *Nat. Commun.* **12**, 2236 (2021).

43. X. Ji, F. A. Barbosa, S. P. Roberts, A. Dutt, J. Cardenas, Y. Okawachi, A. Bryant, A. L. Gaeta, M. Lipson, Ultra-low-loss on-chip resonators with sub-milliwatt parametric oscillation threshold. *Optica* **4**, 619–624 (2017).

44. www.eospace.com/phase modulator.

45. www.thorlabs.com/thorproduct.cfm?partnumber=LNP4216.
